# Interactive Models in Synthetic Biology: Exploring Biological and Cognitive Inter-Identities

**DOI:** 10.3389/fpsyg.2020.00682

**Published:** 2020-04-15

**Authors:** Leonardo Bich

**Affiliations:** IAS-Research Centre for Life, Mind and Society, Department of Logic and Philosophy of Science, University of the Basque Country (UPV/EHU), San Sebastián, Spain

**Keywords:** regulation, synthetic models, communication, minimal cognition, criteria of relevance, criteria of evaluation, Turing test

## Abstract

The aim of this article is to investigate the relevance and implications of synthetic models for the study of the interactive dimension of minimal life and cognition, by taking into consideration how the use of artificial systems may contribute to an understanding of the way in which interactions may affect or even contribute to shape biological identities. To do so, this article analyzes experimental work in synthetic biology on different types of interactions between artificial and natural systems, more specifically: between protocells and between biological living cells and protocells. It discusses how concepts such as control, cognition, communication can be used to characterize these interactions from a theoretical point of view, which criteria of relevance and evaluation of synthetic models can be applied to these cases, and what are their limits.

## Introduction

The last decade has been characterized by an increased interest in synthetic models of interactive biological phenomena, from the study of properties of collective prebiotic systems in origins of life scenarios^1^ and biological communication,^2^ to the exploration of the possible contributions of Synthetic Biology to research in Artificial Intelligence.^3^

The aims, scope and conceptual foundations of this enterprise are still in course of definition, and this article addresses some of the main conceptual issues raised by it. It focuses on how synthetic biology can contribute to the study of those biological and cognitive phenomena, such as for example communication, that arise in nature from the interaction *between* biological systems. In doing so, it takes into considerations different types of inter-systems interactions studied through synthetic models: structural and (minimally) cognitive. Structural interactions are defined as those interactions that directly affect the constitution of the system; cognitive ones as those interactions that are mediated by sensory–effector regulatory mechanisms.

This article discusses what kind of impact these interactions have on the systems involved, and whether and how they affect the identities of such systems. The “identity” of the system, in this context, is defined by the specific organization that characterizes it, and which is kept invariant despite the structural variations that may affect the components (Maturana and Varela, 1980).^4^ As part of the research topic “Inter-identities’ in Life, Mind, and Society,” this article analyzes how identities in interaction can be studied by means of synthetic biology. It is important to emphasize that synthetic models raise closely interconnected theoretical and epistemological questions in relation to interactive identities. The theoretical question is twofold. On the one hand it concerns the relationship between the identity of a system and its interactive capabilities, i.e., how the organization of a system specifies the types of interactions the system can participate in. On the other hand, it concerns whether and how interactions between systems may change their intrinsic properties. Yet analyzing models cannot be separated from the problem of assessing their relevance for studying cases of interacting identities in nature, and from the complex question of how to evaluate whether the models are successful or not in contributing to an understanding of these phenomena.

Accordingly, this article analyzes and discusses four different issues regarding interactive synthetic models: theoretical grounding, criteria of relevance, realization, and strategies of evaluation. To address the issue of the *theoretical grounding* of interactive synthetic models, in section “Theoretical Grounding: Structural and Cognitive Interactions” it provides a characterization of interactions at the specific level which is relevant for synthetic biology. Of particular interest in this context are those interactive properties that can be realized in synthetic biological systems through biochemical mechanisms. The paper adopts a specific theoretical account of minimal cognition based on the notion of biological autonomy to distinguish between structural and cognitive interactions and to provide theoretical tools for their synthetic investigation. It applies this framework to the analysis of those synthetic models that explore interactions – e.g., communication – *between systems* (i.e., between artificial systems, and between artificial and living systems), rather than between one system and its generic environment, and puts into evidence the main challenges they face.

On the basis of the theoretical framework proposed, section “Criteria of Relevance of Interactive Synthetic Models” provides an epistemological analysis of the *criteria of relevance* of synthetic models, and discusses how they apply to this specific scenario in which the focus is on structural and cognitive interactions.

The third issue addressed in this article is the *realization* of interactive synthetic models. The theoretical and epistemological tools developed in sections “Theoretical Grounding: Structural and Cognitive Interactions” and “Criteria of Relevance of Interactive Synthetic Models,” are employed in section “The Realization of Interactive Synthetic Models” to discuss experimental examples of two classes of interactive synthetic models, which cover different types of interacting entities at distinct levels of organization:

(1) Interactions between protocells.
(2) Communication between living cells and protocells.

Finally, section “Conclusions: Evaluation Strategies” discusses limits and merits of three different *strategies of evaluation* of interactive synthetic models: Turing tests, demarcating definitions, and operational approaches. It argues that operational evaluation strategies are the most suitable with regards to the types of phenomena described and theoretical questions addressed by interactive models.

## Theoretical Grounding: Structural and Cognitive Interactions

In order to discuss the contributions of synthetic models to the study of biological and cognitive interactive phenomena, a theoretical framework is required. The framework adopted in this article is the organizational one, based on the notion of autonomy (Varela, 1979; Maturana and Varela, 1980; Kauffman, 2000; Moreno and Mossio, 2015). The notion of autonomy has been often applied in Synthetic Biology to study origins of life, minimal life (Luisi and Varela, 1989; Ruiz-Mirazo and Moreno, 2004; Luisi, 2006), and minimal cognition (Bourgine and Stewart, 2004; Stano et al., 2012; Bich and Moreno, 2016). This framework has also been used to develop epistemological tools to analyze synthetic models (Damiano et al., 2011).

According to this framework, a biological organization – such as a bacterium – is autonomous because it is capable of producing its own functional components and maintaining itself in far from equilibrium conditions. A living system cannot exist unless it maintains a continuous coupling with its environment, made possible by an internal dynamical variability, which enables the system to exert a fine-tuned control upon the exchanges of matter and energy with the surroundings and bring forth different viable responses to a variety of environmental perturbations.

In this scenario, *the identity of the system is identified with its self-maintaining autonomous organization*: the dimension of the system that is maintained invariant despite the continuous structural variations that occur as its components are synthesized, transformed and degraded and its dynamics are perturbed by interactions with the environment and with other autonomous systems. To analyze interacting identities from this perspective, it is necessary to consider (1) what types of interactions between biological autonomous systems are enabled by their distinctive organizations and (2) how such interactions may affect the identities of the interacting systems.

Let us start by considering how interactions are characterized within this framework. Traditional work on biological autonomy – in particular Piaget’s (1967) and Maturana and Varela’s (1980) – and more recent contributions (Stewart, 1992; Bourgine and Stewart, 2004) have defended the thesis, also known as the “Life = Cognition Thesis” (Heschl, 1990), according to which the interactive dimension of life is related to, or coincides with cognition. In this perspective cognition is defined as the interactions with the environment and the relative internal modifications that an organism can undergo without losing its identity (see also Bitbol and Luisi, 2004; Damiano and Luisi, 2010; Bich and Damiano, 2012). The thesis is based on the implicit assumption that living systems are adaptive, in the sense that they are capable of interacting viably with a changing environment by modifying their internal structures. Whereas a perturbation is just external influence for physical systems, living systems, instead, adaptively integrate, and transform it into a “meaningful interpretation” (Heschl, 1990, p. 13). However, the identification of minimal cognition with *all* the interactions an organism can undergo without losing its identity^5^ can be criticized as too broad, on the grounds that it would include: (1) cases of mere covariance between system and environment; (2) the metabolizing of environmental substrates; (3) purely mechanical interactions that cause changes in the systems involved (see also Bich and Moreno, 2016).

To provide a more precise account of the types of interactions biological autonomous systems can experience, and whether or not they may be considered as minimally cognitive, let us first consider which internal changes a minimal living system can undergo without losing its identity while interacting with the environment. According to recent developments of the autonomy framework, the internal changes an autonomous biological system can undergo fall into two general categories: *dynamic stability* and *regulation* (Bich et al., 2016). Dynamic stability is an internal response to interactions with the environment instantiated, for example, by the basic (first-order) metabolic network of processes of production of the components (e.g., enzymes catalyzing metabolic reactions) that realize the living system. It is a general network property: variations in a given process or subsystem can propagate throughout the living system, producing the change of one or several other processes which, in turn, compensate for the initial one. As a result, the system can be regarded as stable. At the level of the basic first-order metabolic regime of self-production and self-maintenance of the system, the compensation for perturbations occurs through reciprocal adjustments between the activity of components, such as enzymes, involved in processes of production, usually stoichiometrically, depending on changes in concentrations of metabolites. These internal changes support a type of interaction with the environment that relies on the structural plasticity of the system. These *structural interactions* are governed by first-order mechanisms and are the most basic responses a system can bring forth while interacting with its environment.

The second type of response falls under the category of regulation, and requires, instead, a more complex, hierarchical architecture. It consists in the capability to selectively switch between different basic (first-order) regimes of self-maintenance in response to interactions with the environment or to internal variations, due to the action of dedicated (second-order) subsystems that are specifically sensitive to these variations. When regulation is at work, the internal dynamics of the basic first-order regime of self-production of the living system are modulated by specialized second-order sensory–effector mechanisms. The activation of these mechanism is triggered by external or internal variations, and as a result of their regulatory activity, the system is able to maintain its viability. Minimal examples of regulatory mechanisms are the *lac*-operon, the tryptophan operon, or the chemotactic signal transduction pathway, to cite just a few well known cases of modulation of the basic (first-order) metabolic and agential dynamics of a system. The distinctive feature of regulatory mechanisms is that as second-order control subsystems they are operationally decoupled from the first-order regime they regulate.^6^

After distinguishing these two general types of adaptive^7^ interactions, based on dynamic stability and regulation respectively, it is possible to discuss whether or not they can ground cognitive properties, as claimed by the L = C thesis. Let us consider a distinctive feature of cognition, which can be realized by minimal living systems. It is the capability to identify or distinguish between some features of their interaction with the environment (for example, the sensing of variations in boundary conditions, concentrations of nutrients, and presence of predators) and to act accordingly, in such a way as to maintain viability. As argued in Bich and Moreno (2016), these cognitive capabilities, at a minimal level, necessarily require regulatory mechanisms in the context of a self-maintaining biological system.

Structural interactions, sustained by distributed responses in a regime of dynamic stability, cannot account for this distinctively cognitive capacity to make meaningful distinctions and to act accordingly. In this type of interactions perturbations just trigger internal changes that are percolated through the system by means of reciprocal adjustments between the activities of the components of the first-order network: the environment is only a source of generic noise.

The requirement for cognition can be met, instead, in presence of dedicated regulatory mechanisms, endowed with sensory–effector capabilities, whose response is the result of the evaluation of perturbations. By means of second-order, operationally decoupled regulatory mechanisms, the system establishes some categories in the environment (sensory capability), and employs them to modulate its own internal dynamics in a viable way (effector capability) in such a way that the system maintains its identity. The organism does things according to what it distinguishes in its interactions with the environment. It modulates its own constitutive dynamics coherently with the variations that activated the regulatory mechanisms, and as a result it maintains its viability in the changing environment: for example, it changes direction of movement or synthesizes a new set of enzymes that allows it to metabolize different substances. In this way, perturbations achieve an endogenous, operational meaningful, significance for the system, which can be considered cognitive in a minimal sense. According to this perspective, therefore, the adaptive behavior of minimal organisms such as bacteria is already cognitive, but only insofar as it is supported by regulatory mechanisms.^8^

From this theoretical standpoint it is possible to discriminate between cognitive and non-cognitive (structural) adaptive interactions. The advantage of this framework is that it provides conceptual tools that can be operationally applied in the biochemical domain to study different minimal biological interactions – structural and cognitive – by means of synthetic models. An example of synthetic realization of cognitive adaptive properties is the implementation of biochemical sensory–effector regulatory mechanisms in protocells or semisynthetic cells (e.g., compartmentalized riboswitches) (Martini and Mansy, 2011).

The synthetic investigation of structural and cognitive interactions and of their relationship with the identity of the systems involved can be pursued in two ways. One is to focus on one artificial system and to analyze how it interacts adaptively *with its environment* by means of either distributed or self-regulatory mechanisms (see Bich and Moreno, 2016). The other way, which is discussed in the rest of this article, is to explore the possibilities opened by the adaptive interactions *between systems* (artificial systems or artificial systems with biological ones). It is inspired by a long research tradition in cybernetics and systems theory opened by the pioneering work carried out by Ashby (1954, 1956), Beer (1972), and Pask (1975), among others –, who had been focusing on the interactive dynamics of systems of different nature (e.g., computational, biological, social, etc.) endowed with self-regulatory mechanisms.^9^

## Criteria of Relevance of Interactive Synthetic Models

In biological systems, constitutive and regulatory adaptive mechanisms – which underlie structural and cognitive interactions, respectively – are endogenously produced and maintained. With their activity, they functionally contribute to the existence of the same system that produces them.^10^ The synthetic modeling of these interactions can be pursued in two different ways. The first consists in the realization of full-fledged interactive systems. It requires integrating regulatory mechanisms into a whole regime of self-production and self-maintenance. However, this approach is especially problematic to pursue in protocells, due to difficulties in realizing a full self-maintaining metabolism (Rampioni et al., 2014). Therefore, at the current state of the art, it is pursued by using metabolically active biological cells as starting points. The second approach aims to realize *life-like* adaptive systems in order to investigate by means of artificial systems specific aspects that are of special interest for a better understanding of structural or minimally cognitive interactions.

Before analyzing how these approaches can be pursued in synthetic biology to investigate interactive identities and how to evaluate the results obtained, let us take an epistemological step and discuss the criteria to assess the criteria of *relevance* of interactive synthetic models to the study these interactions. As argued by Damiano et al. (2011) and Damiano and Cañamero (2010, 2012), one of the goals of a *theoretically inspired* synthetic approach is to create trans-disciplinary exchanges with natural sciences that inspire naturally based technologies, and provide new insights into natural phenomena by means of artificial systems.^11^ In this context, the synthetic approach can allow to experimentally explore aspects of life and cognition that are not (easily) accessible by directly investigating natural systems. It can do so by actually constructing the object of study, an alternative biological or proto-biological system, and study the properties and behaviors it exhibits.

What is the relevance of synthetic models for the scientific investigation of the target interactive biological or cognitive phenomena? Damiano et al. (2011) distinguish two main types of relevance: *phenomenological* and *organizational*. A synthetic model is *phenomenologically relevant* if it produces, according to explicit parameters, the same phenomenology as a living or cognitive system, regardless of the underlying mechanisms, which can be very different. In the case of minimal cognition, for example, a model is relevant at the phenomenological level if it produces the same behavior as a cognitive system, or it engages in similar interactive dynamics.

**FIGURE 1:**
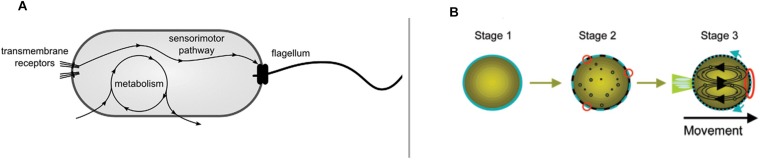
Systems capable of chemotactic behavior by means of radically different mechanisms: **(A)** The sensory motor pathway of a chemotactic bacterium and its relative independence from metabolism (from Egbert et al., 2010, *reproduced under the terms of the Creative Commons Attribution License*); and **(B)** a self-propelled droplet (*reproduced with permission from* Hanczyc et al., 2007). *Copyright (2007) American Chemical Society*.

A paradigmatic case of phenomenological relevance of interactive synthetic models is constituted by relatively simple artificial (chemical) systems such as self-propelled oil droplets capable of chemotaxis (Hanczyc and Ikegami, 2010) (Figure 1B). Chemotaxis is a behavior also exhibited by biological systems such as bacteria (Figure 1A), and it is often considered a hallmark of minimal cognition (van Duijn et al., 2006; Bich and Moreno, 2016).

Both systems, bacteria and droplets, show a similar phenomenology: they are capable of moving and following a chemical gradient. Yet, despite the similarity of behavior, the way behavior itself is generated is very different in the two cases. Self-propelled droplets do not self-maintain like living cells do. The movement of droplets does not rely on nutrients encountered while exploring the environment, but they move by consuming the internal propeller (oleic anhydride) that is already available. In turn, the movement does not contribute to the existence and maintenance of the droplet as it does, instead, in bacteria. There is no internal organizational differentiation (no modular subsystems) within the droplets. Bacteria, instead, exhibit a complex regulatory mechanism (the signal transduction pathway) that modulates a motor system, plus a decoupled metabolism which provides movement and energy to the system. Finally, the direction of the movement of the droplet is directly controlled by the gradient rather than, like in bacteria, by the specific organization of a sensory–effector regulatory subsystem.

While giving precious information on the interactive dynamics of the entities involved, synthetic models that exhibit phenomenological relevance alone – insofar as they provide a point of view that is external to the system and focused on a behavioral description – fail to account for the distinctive features of minimally interactive systems and to discriminate between different types of interactions. For example, if the defining features of minimal cognition are identified in self-regulatory biochemical mechanisms subject to a regime of self-maintenance, then they need to be investigated at a different level of analysis, internal to the system. The same holds for structural interactions, which rely on distributed compensatory mechanisms. Modeling these interactions, therefore, requires different types of synthetic models, whose relevance lies in the organizational, instead of phenomenological, dimension.

A synthetic model is *organizationally relevant* if it realizes the same organization as the living or cognitive system which is the object of investigation (Damiano et al., 2011); in other words, if it realizes the same or a very similar identity. This criterion of relevance focuses the attention on the way components and processes are wired together, according to a specific theory of life and/or cognition. The primary target is not the features of a phenomenon or behavior, but how it is generated. However, achieving organizational relevance does not imply that there should be a strict correspondence between the specific components of the model and the target system. The same type of organization (understood as the topology of relations between components) can be realized by different structures (Maturana and Varela, 1980). Synthetic biologists, therefore, can use whichever minimal biochemical tools they have available to achieve their modeling goal and produce organizationally relevant systems, without the need to reproduce the exact composition of current biological systems, which is the result of a long, complex, and not yet well understood historical process of prebiotic and biological evolution.

The ultimate target for organizationally relevant synthetic models in the framework of biological autonomy is to realize self-producing and self-maintaining protocells capable of interacting adaptively with their environment. Another way to develop organizationally relevant models consists in narrowing down the scope of the model and investigating a specific property or capability of a living of cognitive system, instead of the whole, integrated, entity. In such cases a model achieves what can be called a *mechanism-related organizational relevance* by realizing the same underlying mechanism responsible for a specific behavior or phenomenon.

**FIGURE 2:**
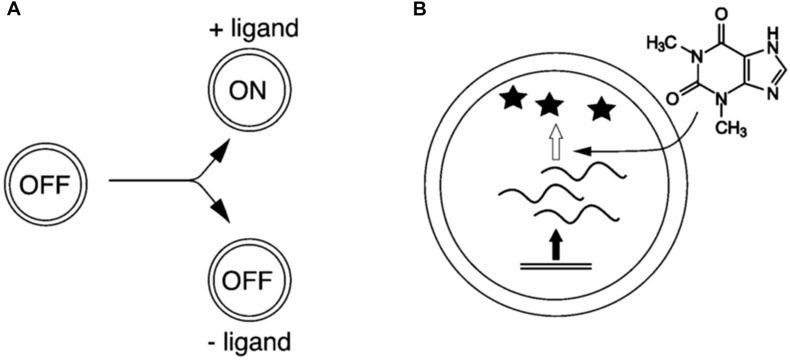
Compartmentalized, cell-like systems that sense and respond to their environments through riboswitch activity. **(A)** The presence of an extravesicular ligand converts the cell-like system from the OFF-state to the ON-state. **(B)** RNA (squiggly line) is transcribed from DNA (double line). RNA is only translated into protein (star) in the presence of the activator ligand, which in this case is theophylline (Martini and Mansy, 2011, p. 10734; *reproduced by permission of The Royal Society of Chemistry*).

An example of this approach in relation to the study minimal cognition is the case of sensory–effector mechanisms implemented in protocells, which allow protocells to sense the environment and change their internal activity accordingly. Such mechanisms have been realized by endowing protocells with riboswitches. As shown experimentally by Martini and Mansy (2011), protocells enclosing riboswitches can indeed sense specific molecules and respond to them by activating DNA-transcription mechanisms (Figure 2). This approach allows investigating specific mechanisms by means of synthetic models, without incurring into the overwhelming difficulties of realizing fully fledged artificial autonomous systems. While at the moment this model does not provide direct insight upon the contribution of such adaptive mechanisms to the internal dynamics and maintenance of the system, it can be particularly interesting from a point of view focused on interactions, as a starting point to study the roots of minimal interactive capabilities of biological systems by modeling their underlying adaptive mechanisms.

Organizational relevance refers to the capability of models to account for the identity of the natural systems under scrutiny. To study interactive identities synthetic models should include those types of internal mechanisms responsible for adaptive structural or cognitive adaptive interactions. Yet the target of these models is not adaptivity alone, but also how biological or life-like systems are capable of *adaptively interacting among themselves*. Therefore, in order to study interactive identities, also an external (inter-system) point of view is needed, capable of specifically taking into account the features of the interactions that those systems undergo without losing their viability, and how the consequent internal modifications in turn affect their identities.

It follows that relevant interactive synthetic models should satisfy criteria belonging to both the general classes introduced by Damiano and collaborators (Damiano et al., 2011). They need to satisfy organizational criteria of relevance for regulatory mechanisms or structural plasticity: either full-fledged, within self-maintaining systems, or mechanism-related, within non-autonomous protocells. But they need to satisfy the criterion of *phenomenological interactive relevance* as well, to be useful tools to explore some aspects of the natural phenomenology of interacting natural systems. To achieve this type of relevance, they need to exhibit sustained successful interactions: that is, viable and leading to adaptive modifications (new responses).

## The Realization of Interactive Synthetic Models

To summarize the previous steps, structural interactions in biological systems rely on distributed responses enabled by the plasticity of the basic network of first-order metabolic components, while minimally cognitive ones rely on dedicated self-regulatory mechanisms. This section will focus on two types of interactive synthetic models: interactions between protocells, and communication between protocells and natural cells. The aim of these groups of examples is to shed light on different aspects of interactive synthetic models – respectively, structural and cognitive – in order to discuss: (1) their theoretical pertinence in modeling interactive identities; (2) how the identities of the systems involved are affected; and (3) whether or not they satisfy the different criteria of relevance relative to the type of interactive phenomenon investigated and the specific theoretical aims of the models.

### Constitutive Interaction Between Protocells

The most basic explorations of interactive phenomena by means of synthetic models are represented by the study of interactions between entirely synthetic systems. The focus is on life-like collective phenomena at the level of protocells, designed with the aim to establish how deeply these phenomena are rooted in prebiotic evolution and what role they might have played in the origins of life.

**FIGURE 3:**
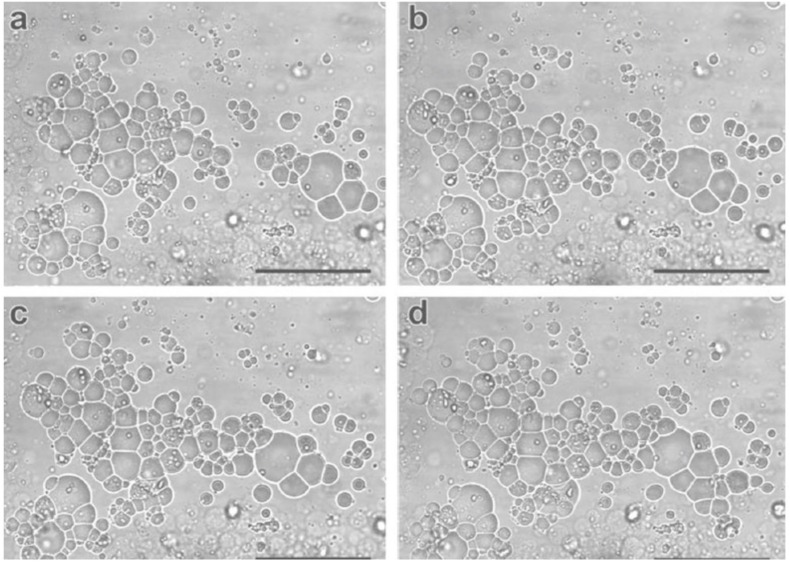
Interactions between protocells. Reciprocal attraction and progressive accretion **(a–d)** in colonies of giant vesicles (Carrara et al., 2012; *reproduced by permission of John Wiley & Sons, Inc.*).

Let us consider two cases: protocell colonies and protocell predation. Experiments with compartmentalized systems, such as liposomes, exhibit problems related to the low permeability of these vesicles, making the incorporation of materials within individual vesicles, or the exchange of material between them very difficult. That is an important issue for the study of the origins of life, insofar as it represents a serious obstacle to the incorporation and exchange of substrates necessary for the beginning of a proto metabolism. In addition to these experimental issues related to the construction and study of individual vesicles, the fact that unicellular prokaryotic organisms live in colonies has given support to the hypothesis that life might have arisen collectively from the cooperation between prebiotic systems, and that important steps in prebiotic evolutions has been enabled by collective phenomena. To pursue this lead, Carrara et al. (2012) have investigated the properties of colonies of giant oleate-based vesicles with negatively charged membranes (Figure 3). The results show that these vesicles form physically stable colonies, attach to solid substrates and exhibit the capability to attract positively charged compounds. Importantly, if compared to individual vesicles, those vesicles belonging to colonies exhibit increased permeability and the capability to incorporate solutes and even larger compounds, which can attach to the membrane and slowly penetrate it without causing its rupture. These phenomena support a possible scenario of the origin of metabolism where externally formed polymers are captured by primitive compartments. In addition, also colony accretion, vesicle fusion, and exchange of material between vesicles have been observed.

The type of interaction modeled by the synthetic systems is structural, insofar as no regulatory mechanisms are employed but instead the physical properties of the subsystems are involved. It is important to point out that the interactive artificial vesicles give rise to new capabilities owing to the collective nature of the phenomenon.

The synthetic interactive model exhibits phenomenological relevance insofar as it shows new biologically relevant properties – such as collective behaviors and attachment to substrates, exchange of materials between vesicles, etc. – and gives rise to a successful interaction (stable and sustained). Organizationally speaking, the system does not exhibit either internal differentiation or specialized mechanisms controlling the interaction. Yet the capability of vesicles belonging to colonies to attract and incorporate molecules can be interpreted as organizationally relevant from a constitutive point of view within an origin of life scenario, as a possible step toward the emergence of metabolism.

To identify the implications of this type of model for our understanding of interactive identities it is necessary to consider what types of interactions are enabled by the distinctive organizations of the systems involved and how such interactions may affect the identities of the interacting systems. The type of structural interaction taking place between these oleate-based vesicles is made possible by the electric charge of their membranes, which allow attraction of other vesicles and chemical compounds. The physical properties of the systems involved are affected and modified by such interactions, and new capabilities are acquired at the collective level, although not directly affecting the very (simple) organization of the vesicles. Yet the resulting incorporation of new material due to the increased permeability has the potentiality for triggering organizational changes. New molecules capable of entering new interactions and playing a functional role in the vesicle could trigger transitions toward new and more complex identities, for example, by catalyzing metabolic reactions within the vesicles, or inserting themselves into the membrane and give rise to primitive pores or channels.

The second example of synthetic model of a collective behavior focuses on protocell predation, with the aim to explore its possible role in prebiotic evolution (Qiao et al., 2017). The investigation of this type of interaction is also based on the assumption regarding the importance of collective phenomena at the origins of life: that life does not occur in isolation, and that living systems compete for resources or directly predate on one another (Mansy, 2017). Two types of protocells are included, which harbor different cargo molecules such as protease, DNA and sugars. They are characterized by different compositions of their compartments, and carry opposite electrostatic charges to facilitate interactions. The predator protocells are constituted by coacervates, which contain a protein -degrading enzyme protease. The preys are proteinosomes, i.e., protocells enclosed by a proteic compartment, and harboring DNA and sugars. Once the two types of cells interact, the coacervates digest the proteic membrane of the preys and assimilate their internal DNA and sugar. Then, the presence of different internal molecules in the predator can be potentially selected by the environment. However, further effects of predation beyond the digestion of the prey and capture of its contents, which are structural interactions, go beyond the scope of this synthetic model. It does not include specialized internal adaptive mechanisms – such as sensory-motor regulatory mechanisms usually associated to preying and escaping – and does not give rise to phenomena such as population growth and inheritance. The interaction is not sustained, but results in the metabolic absorption of the contents of the prey protocells. In sum, this model cannot account for coordinated behaviors between predators and preys, and for oscillatory populations such as those exhibited by traditional Lotka–Volterra predator–prey models. Therefore, while describing a model of a possible structural interaction which may radically modify the identity of the systems involved, it exhibits low phenomenological and organizational relevance *as a model of the specific phenomenon of predation*.

The organization of the systems involved enables interactions based on reciprocal attraction of protocells due to opposite electrostatic charges. In addition to that, the predator protocells contain protease enzymes that allow the digestion of the proteic membranes of the preys. The effects of the interaction on the identities of the two types of systems involved are different. The preys disappear, while the predators acquire new components (DNA and sugars). Yet the interaction has no effect on the identity of the predator unless the new components acquire a functional role within it, thus modifying its organization.

These models provide important and original insights into the interactive origin of some constitutive features of protocells in the prebiotic world, such as the presence of internal molecules despite the absence of full-fledged membrane channels. Moreover, both models employ electrostatically charged membranes to facilitate structural interactions in protocells that lack sensors. The focus here is specifically on composition and structural properties of protocells. For these very reasons the models do not provide information on more complex adaptive (structural and cognitive) properties, as the protocells employed in these synthetic models do not exhibit internal differentiation and regulatory mechanisms. As models of interactive capabilities, they exhibit phenomenological relevance, insofar as they carry out interactions that affect the constitutive identity of the system. Instead, they exhibit low organizational relevance, as in addition to not realizing self-maintenance, they do not employ life-like interactive biochemical mechanisms, but rely on opposite membrane charges. Nevertheless, these interactions may affect the structures of the systems involved and have the potential to modify their identities by triggering organizational transitions.

A possible further step into the investigation of protocell interactions would be to increase the complexity of the systems involved, in such a way as to model minimally cognitive interactions. One possibility is to add basic sensory–effector mechanisms such as riboswitches – whose effects change the protocells membrane properties or behaviors toward other protocells as a result of sensing the state of latter – and observe the resulting collective dynamics and the potential emergence of self-organized patterns of interactions.

### Communication Between Living Cells and Protocells

More complex types of interactions engage biological cells and protocells. They rely on signal exchanges, enabled by sensory–effector mechanisms with activation of regulatory responses. A thriving line of investigation in this branch of synthetic biology focuses on the phenomenon of biological communication for technological^12^ and theoretical purposes.^13^

**FIGURE 4:**
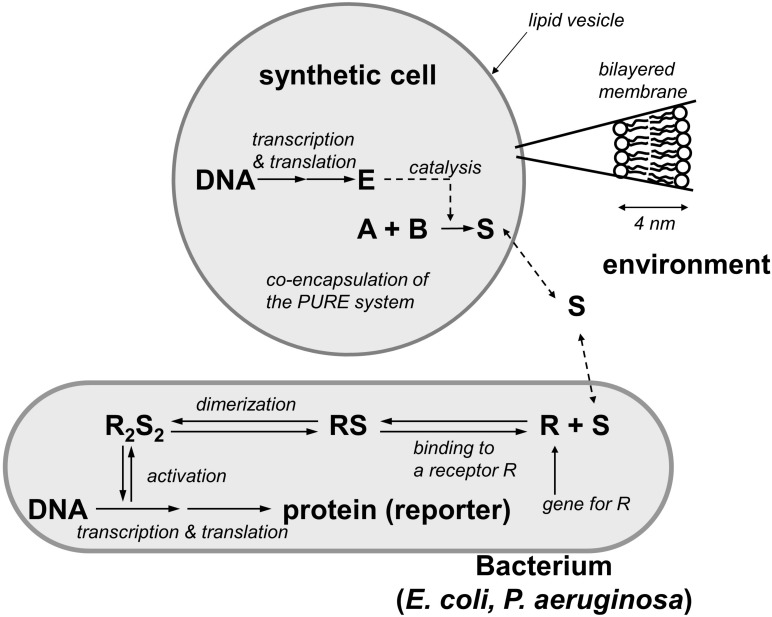
Simplified model of an artificial cell synthesizing signal molecules that can be sensed by natural cells: a liposome contains DNA and a transcription-translation machinery (PURE system). An enzyme E is synthesized and catalyzes the reaction that produces a signal molecule S from the substrates A and B (redrawn with modifications by Pasquale Stano from Rampioni et al., 2014).

The basic idea underlying theoretical research on synthetic communication is to design protocells that send signals and trigger responses in living cells such as bacteria. An example of this approach is provided by Rampioni et al.’s (2014) simplified model of synthetic cell sending signals to a natural cell. This study evaluates the realizability of protocells with effector capabilities. The idea is to include into liposomes the biomolecular machinery necessary to produce signal molecules (e.g., *N*-acyl-homoserine lactones) that, sensed by the receptor of a natural cell, can trigger processes of protein synthesis in the cell (Figure 4).

A more recent model designed by Lentini et al. (2017) attempts to realize a two-way communication. Endowing protocells with the capability to sense (activating transcriptional regulatory binding sites within the protocell) and produce quorum molecules, it makes them able to interact with bacteria and even to interfere with quorum sensing mechanisms in the latter.

The complexity of these models raises several theoretical and epistemological issues when considering their phenomenological and organizational relevance, and more generally on the enterprise of investigating biological communication by means of synthetic biology. The models exhibit successful interactive capabilities through the activity of biochemical regulatory mechanisms. In particular, the second model achieves (mechanism-related) organizational relevance by realizing a whole sensory–effector mechanism, while the first model focuses on effector mechanisms.

Lentini et al.’s (2017) is a model of minimally cognitive-like interactions, supported by regulatory mechanisms. Yet it is not necessarily a model of biological communication. To make this point clear, let us consider a well-known biological case of interactions that are supported by sensory–effector mechanisms, like in the case described in Lentini et al. (2017), but are not considered as a case of communication: the interaction between a lion and a gazelle. It exhibits some analogies to the model just described. The lion sees the gazelle and start chasing it. The gazelle, seeing or hearing the lion approaching, starts running to escape. Then the lion adjusts its course to the new path and speed of the gazelle, etc. This is a case of cognitive interaction in which two biological systems realize a form of coordinated behavior supported by their own internal regulatory mechanisms. Nevertheless, it is clearly not a case of communication.

These synthetic models face a demarcation problem. They aim to realize communication by means of coordinated behaviors, but the way the phenomenon of communication is framed, would include also non-communicative cognitive interactions such as the one exemplified by the lion chasing a gazelle. Hence, they need a different theoretical grounding, based on a conceptual framework of communication that can be operationalized and applied to synthetic biology and can in principle discriminate between communication and other types of cognitive interactions.

The account of biological communication as functional influence seems a good candidate in this regard. In its most general formulation, it characterizes communication as an interaction in which a signal emitted by a sender triggers a change in the behavior of the receiver that is functional for the sender itself. The functional dimension is essential, as it allows distinguishing cases of communication from other interactions that trigger mutual changes in the systems involved. In the case of the lion-gazelle predator–prey system, for example, it is possible to argue that the interactions trigger changes in the behaviors of the two systems, but not that the noise made by the lion has the *function of* triggering the escape of the gazelle (Bich and Frick, 2018; Frick, 2019).

A remark is due. This specific account of communication was introduced by Dawkins and Krebs (1978) in an evolutionary framework, according to which what is functional for the sender, is interpreted in terms of adaptations: the signal is a functional trait because it allowed the ancestors of the sender to survive. Yet, in this specific form, it cannot be applied to artificial systems that are the result of synthetic biology rather than evolution by natural selection. Focusing on evolutionary adaptations, it does not support questions about communicative phenomena happening *here and now*.

To be applied in synthetic biology, this account and its functional dimension can be reformulated in organizational terms,^14^ in which to say that a signal is functional, specifically means that it contributes to the maintenance of the current organization of the sender (Frick et al., 2019). In this form it can be applied in principle to artificial systems and can provide the criteria needed to overcome the demarcation problem, by discriminating between communication and other minimally cognitive interactive phenomena in synthetic models.

The *organizational-influence approach* provides an *operational* characterization of communication in terms of sensory–effector regulatory interactions, which can be applied to the design of those mechanisms and phenomena specifically studied through synthetic models (Bich and Frick, 2018). Let us consider two systems: a sender A and a receiver B. The sensory parts of the regulatory mechanisms of A are activated by specific features of their interaction with the environment, and their effector parts modulate the internal dynamics of the system. The modified system A produces a signal, which triggers a regulatory action in B, the receiver, which changes its behavior. The new behavior of B is functional for the sender in the sense that it contributes to the maintenance of A in the context that activated the regulatory action in A. According to this approach, the interaction between A and B can be said to be both cognitive (it employs sensory–effector regulatory mechanisms) and communicative (it is functional for the sender). In this case the identities of the systems are not structurally modified like in the examples discussed in section “Constitutive Interaction Between Protocells.” They are supported and maintained by recruiting the functional contributions from the receivers.

**FIGURE 5:**
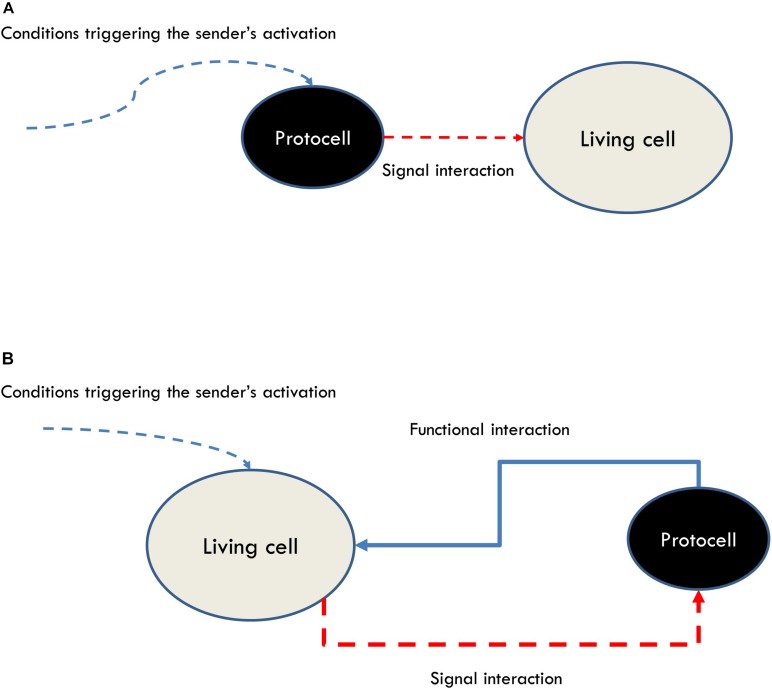
**(A)** A broad notion of communication modeled through the interaction of a protocell sending a signal to a living cell. This approach would include phenomena which are not generally accepted as instances of communication. **(B)** A more discriminating, organizational account of communication modeled through a functional loop realized by a living cell interacting with a protocell.

While providing demarcation criteria, this theoretical account does not impose strong operational requirements for the synthetic realization of communication apart from (1) the presence of sensors and effectors (organizational criteria of relevance) and (2) that the operations of the systems involved should exhibit a specific pattern of interactions (which correspond in this case to the phenomenological interactive criteria of relevance) (Figure 5B).

Adopting the organizational-influence account allows reframing the models of communication between protocells and living cells discussed above, in such a way that they can capture the distinctiveness of communicative interactions. This theoretical framework can be operationalized by employing and redesigning, in terms of functional influence, the already available protocells with sensory–effector capabilities. A pertinent remark that can be raised at this point is that protocells are not functional in themselves in the sense that they are not self-maintaining systems. This has two implications. The first is that it is still possible to realize synthetic models that are phenomenologically relevant but that realize only mechanism-related organizational relevance. The second is that “life-like communication” can be explored and evaluated from the point of view of living cells – which exhibit functionality – by making them interact with protocells endowed with sensory–effector regulatory mechanisms. The idea would be to re-design the interaction by realizing systems in which sender cells are capable, through signals, to influence protocells in a way that is functional to the cells.

In sum, adopting a theoretical framework of communication, such as the organizational one, capable of satisfying both demarcation and operationability requirements can provide guidelines for the design of synthetic models aimed at studying the nature and minimal instances of communication as a specific type of cognitive interaction. In particular, it puts into evidence the necessity for modelers to shift their attention from designing protocells that can interact with cells by triggering changes in the latter (Figure 5A), to protocells that can participate in functional loops with cells (Figure 5B). Moreover, the use of these models, besides contributing to an understanding of the most basic features of the phenomenon of communication and its minimal instances in bacteria, can help to identify the distinctive features of (and clarify the differences between) different types of interactions by focusing the attention on the functional relationships between the interactors, like in the case of communication vs predator-prey interactions.

Analyzing these models can also provide important insights into the question of interacting identities. Organizations that include sensory–effector mechanisms, such as the protocells and cells involved in communication, support minimally cognitive interaction, beside structural ones. In the case of communication, the interaction directly contributes to maintain the identity of the sender and to extend its functional boundary outside the system, as the sender recruits external functions by integrating the receiver into a larger functional loop. If compared to the cases of structural interactions between protocells analyzed in section “Constitutive Interaction Between Protocells,” these interactions have potentially a weaker effect on the identities of the systems involved. The difference with the case of protocell predation is evident. In the case of communication interactions are managed by second-order specialized mechanisms, which in turn modulate the internal first-order dynamics, without the latter being directly affected by the features of the interaction itself.

This comparison shows how regulatory mechanisms contribute to enhance the robustness of the system, insofar as they prevent interactions from directly affecting the core of the self-maintaining regime of a system. In addition, it shows on the one hand how regulatory mechanisms make it more difficult to trigger radical organizational transitions in the first-order regime. Yet, on the other hand, it shows how regulatory mechanisms may provide more reliable ways to modify the organization of a system toward increased complexity. A modification of the core constitutive network of the system has a higher risk to drive the system to disruption than to generate novel and more complex functionalities. A modification of the regulatory subsystems that control internal changes (e.g., switches) provides instead more reliable solutions to introduce novel functionalities by acting on relatively independent (decoupled) regulatory switches instead of radically changing the more basic self-maintaining regime of the system (Kirschner et al., 2005).

## Conclusion: Evaluation Strategies

Addressing the problem of the theoretical grounding of synthetic models (see section “Theoretical Grounding: Structural and Cognitive Interactions”), identifying the criteria of relevance of models (see section “Criteria of Relevance of Interactive Synthetic Models”), assessing whether or not they are satisfied when realizing interacting synthetic models and what are the theoretical implications of the models (see section “The Realization of Interactive Synthetic Models”), are important aspects of their design and discussion. They concern the type of contribution a model can provide to the study of a given phenomenon – in this case, structural and cognitive interactions and their implications for the identities of the systems involved – in relation to a given theoretical framework, such as the organizational account based on the notion of biological autonomy.

A further epistemological issue regarding this branch of synthetic biology is whether and how models are successful at what they aim to do: i.e., the problem of evaluation. This is a particularly difficult task, insofar as these models do not exactly aim to describe or represent natural phenomena such as biological interactions, or to develop predictive tools. Synthetic biology combines technological and scientific methodologies, and this mixed nature is reflected in the goals of the models developed (Green, 2017). On the one hand they aim to realize systems that work, without looking for optimal solutions (O’Malley, 2009). On the other hand, they aim to provide insights into, or a better understanding of, certain features of natural systems, and they do so by means of alternative realizations (Damiano and Cañamero, 2012).

How can we assess whether or not an interactive synthetic model is a good model of interactive identities? The dual nature of the goals of synthetic models makes it difficult to evaluate their success, insofar as this task needs to combine two different types of criteria: (1) pragmatic “whatever works” criteria, i.e., the realization of a device that does what it has been designed to do; with (2) theoretical criteria related to the contribution to a better understanding of biological and cognitive phenomena. The latter ones are particularly complex as they need to establish whether an alternative realization provides useful insights into a phenomenon under study (for example, a specific type of interaction between living systems). In order to combine these two types of criteria, evaluations need to take into account all the dimensions analyzed in the previous sections, i.e., theoretical grounding, relevance, and realization.

An ingenious strategy to evaluate synthetic models, by specifically focusing on their interactive dimensions, has been employed in synthetic biology with the introduction of Turing tests for life (or for *life-likeness*). It explicitly aims to provide satisfactory and unbiased criteria for evaluating the results of the synthesis of life-like systems and behaviors. The basic idea is to have the model pass an evaluation according to criteria that are not dependent on external designers or users, but are somehow intrinsic to the domain of the phenomenon modeled (i.e., biological or cognitive). This result can be achieved by having the model system interact with a natural one, which will play the role of the evaluator.

In general, the principle underlying Turing tests is that a model is valid if it cannot be distinguished from the real thing by an appropriate interrogator. In synthetic biology, the main idea is to let living cells be the interrogators, and let them “evaluate” the models by interacting with the artificial cells (Cronin et al., 2006). This interaction can occur, for example, by means of exchanges of signals, which at first sight makes this strategy very suitable to evaluate interactive capabilities such as those analyzed in the case of biological communication. For example, when artificial cells emit signal molecules, if a response is activated in the living cell, the artificial one passes the test for life (-likeness) (Gardner et al., 2009).

However, this type of evaluation strategy exhibits several limitations which make it difficult to assess the epistemic contribution of a synthetic model to the understanding of a biological or cognitive interactive phenomenon. Despite aiming to provide unbiased criteria, intrinsic to the domain of investigation, these Turing tests are still designer-dependent. The first problem is that the scope of the test is ultimately restricted to the “whatever works” criteria. Let us consider the case of an artificially system (a protocell) that should trigger in a living cell (through a signal molecule) the same response that a biological system would. The designer establishes *a priori* that the life-likeness criterion in this case consists in triggering this specific response, and focuses on the molecule that does so. The Turing test for life does not introduce a further designer-independent, intrinsically biological, criterion when employing a biological interrogator. It confirms that the artificial system does what it was designed to do: trigger a given (already known) response. The living cell, for example, would evaluate positively (as life-like) any other source of the same signal, such as the direct administration of the signal molecule by the experimenter, or by an abiotic reaction.

The second limitation is that the test evaluates only phenomenological mimicry. The interrogation is purely behavioral and does not take into consideration the internal mechanisms. This, for example, is not a good way to test for different types of interactive capabilities, whether they are cognitive, i.e., if these are theoretically characterized in terms of regulatory mechanisms or structural, i.e., supported by internal plasticity (distributed compensatory responses). To be more precise, this interrogation only tests the *response to mimicry by the living cell*. It does not test the behavior of the artificial cell itself, insofar as the response depends on the nature of the signal molecule, and not of the source of the signal. This limit is particularly relevant when one of the aims of the synthetic model is to investigate how different types of interaction take place, and how they affect the identities of the systems involved at the level of their internal organization.

Let us discuss how the test applies to the models discussed in section “The Realization of Interactive Synthetic Models,” to assess its practical limits. Considering that the test implies the evaluation of an artificial cells by a natural one, it does not apply to models of interaction between protocells alone (see section “Constitutive Interaction Between Protocells”). It can be applied, instead, to evaluate interactions between artificial and natural cells (see section “Communication Between Living Cells and Protocells”), but with two caveats. In the first place, given its focus on mimicry, it does not allow evaluating organizational relevance. In the second place, if the focus is on communication (between living cells and protocells) defined in terms of functional influence of a sender upon a receiver, what is required to be evaluated as communication is not the presence of a response by cells to signals released from protocells (what the Turing test for life is designed to do). Rather, as discussed in section “Communication Between Living Cells and Protocells” it is how living cells, the senders, change the behavior of (non-self-maintaining) protocells, the receivers, in such a way that the new behavior functional contribute to the maintenance of the senders. Therefore, the test has problems in evaluating the contribution of the model to the understanding of a biological interactive phenomenon such as communication.

Although limited, the Turing test for life was introduced to overcome the problems exhibited by another evaluation tool employed by synthetic biologists, that is, definitions (of life and cognition) (Forlin et al., 2012). The criticisms of definitions as a tool for synthetic biology, among other disciplines, has been motivated by the lack of consensus on a single definition of a given phenomenon such as life or cognition, and the consequent failure in providing precise and universal criteria, often with different definitions used by different research groups in the same field. Such criticisms (Cleland, 2012; Machery, 2012) are based on the implicit assumption that the role of definitions is to demarcate a phenomenon such as life, and to provide an univocal and definitive answer.^15^ In fact that is not their common use, inasmuch as in the practice of synthetic biology, conceptual models and definitions are rarely employed as direct criteria of evaluation, but play a different role as theoretical and heuristic tools that provide guidelines to build models and design experiments.^16^

The limitations of straightforward evaluation tools such as Turing tests and definitions (in the few cases when the latter are used as demarcation tools) leave us with two possible options. The first is to adopt minimal evaluation criteria, restricted to only one type of goal of synthetic models, that is, successful realization: the “whatever works” criterion. The second is to adopt a more complex, though less straightforward, evaluation methodology. A possible evaluation strategy of this latter type would need to consider (at least) the three aspects which have been analyzed in the previous sections. The first is the theoretical pertinence: how models relate to a specific question in the given context of the phenomenon under investigation, in this case the nature of structural and cognitive interactions and their effects on the identities of the systems involved. The second concerns the criteria of relevance that the model needs to satisfy in the light of the question asked. Finally, the third is how the model solves the issue practically.

Let us consider synthetic models of structural and cognitive interactions. Their design requires a hybrid strategy which combines phenomenological-interactive and (mechanism-related) organizational approaches, theoretically grounded in a definition of structural interactions as relying on first-order network properties, involving distributed responses, and cognitive interactions as adaptive capabilities of living systems based on regulatory mechanisms. Their evaluation should take into consideration: (1) how the model relates to a specific question (its *theoretical pertinence*). In this case it relates to the characterization of structural or cognitive interactions in terms of constitutive and regulatory mechanisms, respectively; or, in the case discussed in section “Communication Between Living Cells and Protocells,” how the model relates to a given theoretical account of communication. The theoretical pertinence should include also whether the model can provide insight into the effects of these different types of interactions on the identities of the systems involved; (2) the capability of *integrating two criteria of relevance* to respond to the theoretical question, i.e., phenomenologically interactive and (mechanism-related) organizational ones; (3) *successful design*, i.e., the fact that the synthetic model is capable of producing a sustained interaction between the artificial and natural systems involved.^17^

From this perspective, the Turing test can be seen as a special case of evaluation of successful design, detached from theoretical considerations, restricted to cells-protocells interaction and to a specific type of phenomenological relevance, that is, response to mimicry, with all the limitations discussed before.

This more complex approach, instead, acknowledges the *operational role* of theoretical considerations and of definitions as guidelines for the design of experiments and models (Bich and Green, 2018). In this case concepts and definitions are not used as sources of checklists or tests for life or cognition (demarcation criteria), but in combination with other types of criteria, such as relevance and successful design. What these models tell us, and needs to be taken into consideration in their evaluation, is not only that interactions between protocells or between protocells and living cells are possible, but something more on the phenomena modeled. They can show the limits of some implicit assumptions such as the idea of behavioral coordination in relation to biological communication, and provide critical insights on the effects of different types of interactions upon minimal systems, such as for example the possibility of deep organizational transitions implied by structural interactions and the specific contribution of regulatory mechanisms to robustness.

## Author Contributions

The author confirms being the sole contributor of this work and has approved it for publication.

## Conflict of Interest

The authors declare that the research was conducted in the absence of any commercial or financial relationships that could be construed as a potential conflict of interest.

## Footnotes

1 For example, colonies of giant vesicles (Carrara et al., 2012) and predator–prey interactions in protocells (Qiao et al., 2017).

2 This research line has been focusing on interactions between biological cells and protocells (Gardner et al., 2009; Lentini et al., 2017; Rampioni et al., 2014).

3 This research line has been pursued by focusing either on information technologies that realize computation through bio- chemical systems (Amos et al., 2011), or on biochemically-grounded embodied artificial intelligence (Stano et al., 2018).

4 For the more general philosophical debate on the notion of identity, see Williamson (1990); Lowe (2002), Miller (2010), and Noonan and Curtis (2018).

5 A strong “L = C Thesis”.

6 Regulatory mechanisms are specialized subsystems dedicated to the modulation of the first-order regime they control. The relation between regulator and regulated subsystems, therefore, is asymmetrical. Regulatory subsystems do not operate as nodes of the same basic network of mechanisms of production of components, but exhibit degrees of freedom that are not specified by the dynamics of the regulated subsystems. Such a local independence allows regulatory subsystems to modulate first-order ones in a relatively independent way. See Bich et al. (2016) for more details on the features and requirements for decoupled regulatory mechanisms.

7 “Adaptive” is used in this context as an interaction that triggers a viable response by means of internal changes in the perturbed system.

8 Very different views have been defended with regards to the nature and lower boundaries of minimal cognition. Some cognitive capabilities have been attributed to chemical systems below the threshold of life, like oil droplets, justified on the basis of their chemotactic behavior which mimics that of bacteria (Hanczyc and Ikegami, 2010). Approaches focused on the specific features of the organizations – i.e., minimal biochemical mechanisms – underlying cognitive capabilities, such as chemotaxis and communication, identify cognition at the level of prokaryotes (van Duijn et al., 2006; Bich and Moreno, 2016). Others identify cognition only in organisms with nervous system (Christensen and Hooker, 2000; Barandiaran and Moreno, 2006). See Godfrey-Smith (2016) for a discussion of different accounts of minimal cognition and of the main transitions in the evolution of cognition and subjectivity.

9 See Damiano (2009) and Pickering (2010) for an analysis of this research tradition.

10 This is an important difference with hardware-based artificial systems, in which parts are put together from without, and interact to produce a certain effect without their operations affecting their conditions of existence.

11 See also Pfeifer and Scheier (1999); Ruiz-Mirazo and Moreno (2013), and Green (2017).

12 The aim is to develop systems for the targeted administration of molecules to living cells by means of protocells (LeDuc et al., 2007) or biochemical information technologies (Nakano et al., 2011), among others.

13 The aim in this case is to provide insight into the nature and origin of minimal forms of biological communication by means of artificial systems (Rampioni et al., 2014, 2019).

14 The organizational account defines the function of a trait in terms of contribution to the maintenance of the system that currently harbors and produces that trait (Mossio et al., 2009), rather than in terms of the evolutionary history of the trait.

15 The same can be said about definitions of cognition.

16 Definitions are not necessarily (definitive) answers, but rather ways to formulate precise questions. In the practice of several disciplines such as synthetic biology, artificial life, systems biology, and astrobiology, definition are used as theoretical and epistemic tools that make assumptions explicit, clear, and challengeable, help formulating questions, and suggest what phenomena to look for, or what to realize in the laboratory (Bich and Green, 2018). Definitions of life are not normally used as definitive answers and tools to demarcate life. Even in astrobiology they have been used to design experiments and devices, not as answers about the presence of life.

17 17It might also include whether the model produces new unexpected phenomena: what Damiano et al. (2011) call “progressive phenomenological relevance.”
